# c-Rel Is the Pivotal NF-κB Subunit in Germinal Center Diffuse Large B-Cell Lymphoma: A LYSA Study

**DOI:** 10.3389/fonc.2021.638897

**Published:** 2021-04-20

**Authors:** Nathalie Faumont, Oussama Taoui, Davi Collares, Jean-Philippe Jais, Karen Leroy, Léa Prévaud, Fabrice Jardin, Thierry J. Molina, Christiane Copie-Bergman, Barbara Petit, Marie-Pierre Gourin, Dominique Bordessoule, Danielle Troutaud, Véronique Baud, Jean Feuillard

**Affiliations:** ^1^ CNRS UMR-7276, INSERM U1262, CRIBL, University of Limoges, and Hematology Laboratory of Dupuytren Hospital University Center (CHU) of Limoges, Limoges, France; ^2^ Université de Paris, NF-κappaB, Differentiation and Cancer, Paris, France; ^3^ Biostatistics Department, Imagine Institute, Paris, France; ^4^ UMRS1138, Centre de Recherche des Cordeliers, Paris Descartes University, CARPEM, Department of Genetics and Molecular Biology, Cochin Hospital, Assistance Publique Hôpitaux de Paris, Paris, France; ^5^ Inserm U1245 and Department of Henri-Becquerel Hematology Center and Normandie Univ UNIROUEN, Rouen, France; ^6^ Pathology Department, Necker Hospital, Assistance Publique Hôpitaux de Paris (AP-HP), Paris, France; ^7^ IMRB-Inserm U955, AP-HP Henri-Mondor Hospital, Créteil, France; ^8^ Pathology Department, CHU of Limoges, Limoges, France; ^9^ Regional Reference Structure of Limousin Lymphomas, Clinical Hematology Department, CHU of Limoges, Limoges, France; ^10^ EA3842, CAPTuR, University of Limoges, Limoges, France

**Keywords:** NF-kappaB, DNA binding activity, c-Rel, germinal center B-cell–diffuse large B-cell lymphoma, genetic alterations

## Abstract

Relationships between c-Rel and GCB-DLBCLs remain unclear. We found that strong c-Rel DNA-binding activity was mostly found in GCBs on two independent series of 48 DLBCLs and 66 DLBCLs, the latter issued from the GHEDI series. c-Rel DNA-binding activity was associated with increased *REL* mRNA expression. Extending the study to the whole GHEDI and Lenz DLBCL published series of 202 and 233 cases, it was found that the c-Rel gene expression profile (GEP) overlapped partially (12%) but only with the GCB GEP and not with the GEP of ABC-DLBCLs. Cases with both overexpression of *REL* mRNA and c-Rel GEP were defined as those having a c-Rel signature. These cases were GCBs in 88 and 83% of the GHEDI or Lenz’s DLBCL series respectively. The c-Rel signature was also associated with various recurrent GCB-DLBCL genetic events, including *REL* gains, *BCL2* translocation, *MEF2B*, *EZH2*, *CREBBP*, and *TNFRSF14* mutations and with the EZB GCB genetic subtype. By CGH array, the c-Rel signature was specifically correlated with 2p15-16.1 amplification that includes XPO1, BCL11A, and USP34 and with the 22q11.22 deletion that covers IGLL5 and PRAME. The total number of gene copy number aberrations, so-called genomic imbalance complexity, was decreased in cases with the c-Rel signature. These cases exhibited a better overall survival. Functionally, overexpression of c-Rel induced its constitutive nuclear localization and protected cells against apoptosis while its repression tended to increase cell death. These results show that, clinically and biologically, c-Rel is the pivotal NF-*κ*B subunit in the GCB-DLBCL subgroup. Functionally, c-Rel overexpression could directly promote DLBCL tumorigenesis without need for further activation signals.

## Introduction

Diffuse large B-cell lymphomas (DLBCL) are the most frequent among aggressive non-Hodgkin’s lymphomas (NHLs). DLBCLs are clinically, molecularly, and genetically heterogeneous, suggesting different oncogenic mechanisms. Gene-expression profiling (GEP) studies led to the proposal of two cell-of-origin (COO) molecular subtypes known as germinal center B-cell-like (GCB) DLBCLs, and activated B-cell like (ABC) DLBCLs, with a subset of cases showing an intermediate, unclassifiable phenotype (1). GCB-DLBCLs seem to arise from normal germinal center B-cells, whereas ABC-DLBCLs would originate from post-germinal center activated B-cells arrested before terminal plasma cell differentiation.

When compared to GCB-DLBCLs, several studies have shown that ABC-DLBCLs are the more aggressive subtype with the worst patient outcome (2, 3). ABC-DLBCLs exhibit constitutive activation of the NF-*κ*B pathway that drives tumor proliferation and survival and confers chemotherapy resistance (4). Constitutive activation of NF-*κ*B in ABC-DLBCLs is due to a variety of mutations in NF-*κ*B regulator coding genes, such as *MYD88*, *TNFAIP3* (A20), *CD79A/B*, *CARD11*, *TRAF2*, *TRAF5*, *MAP3K7* (TAK1), or *TNFRSF11A* (RANK) (5).

In contrast to ABC-DLBCLs, GCB-DLBCLs do not exhibit a NF-*κ*B transcriptomic signature (1). GCB-DLBCL is the most frequent DLBCL subtype and, with primary mediastinal B-cell lymphomas (PMBLs), represents almost all DLBCL cases diagnosed in children, adolescents, and young adults (6). Genetic abnormalities strongly associated with GCB-DLBCLs result in epigenetic modifications such as histone methylation or acetylation (*EZH2*, *EP300*, *CREBBP*, *KMT2D*), B-cell migration (*GNA13*, *GNAI2*, *SIPR2*), PI3K/AKT/mTOR pathway activation (*FOXO1*, *SGK1*, *PTEN*), and immune-regulation (*TNFRSF14*) (5). Additionally, the t(14;18) translocation that brings *BCL2* under the control of IGH locus regulatory regions is detected in about 30% of GCB-DLBCLs but is rarely found in ABC-DLBCLs (7). Despite being reported at variable frequencies ranging 15 to 37%, the *REL* locus (2p16 region) gains including amplification (≥four copies) are among the most frequently observed alterations in the GCB subtype (8). *REL* gains are also recurrent gene abnormalities in other hematopoietic cancers such as classical Hodgkin’s lymphoma, follicular lymphoma, MALT lymphoma, Burkitt’s lymphoma, and PMBL.

The *REL* gene encodes the c-Rel subunit of NF-*κ*B. c-Rel was first identified as a cellular homolog of the avian retroviral oncoprotein v-Rel, which rapidly causes fatal lymphoid cell tumors in young birds (9). Like the other members of the NF-*κ*B family, c-Rel is characterized by the presence of a highly conserved amino-terminal domain so called RHD (*Rel Homology Domain*) responsible for dimerization, DNA-binding, and I*κ*B inhibitory protein binding. c-Rel is the only member of the NF-*κ*B family that can transform avian lymphoid cells *in vitro* (10). Artificial mutants of c-Rel indicate that the transactivation domain in the carboxy-terminal part is responsible for this transforming potential (11).

The role of c-Rel is important in many aspects of lymphoid cell function. Expression of c-Rel is ubiquitous in B-cells and increases during B-cell development, particularly in germinal center B-cells (12). Experimental ectopic c-Rel expression blocks plasma cell differentiation by inhibiting the expression of the transcription factor Blimp1 (13). Reciprocally, c-Rel is repressed by Blimp1 through its binding to the *REL* locus when the plasma cell differentiation process is engaged (13). Transgenic mice lacking c-Rel develop normally without effects on hematopoietic bone marrow development, but exhibit numerous peripheral immunological defects, including reduced proliferation and activation of mature B-cells in response to mitogenic stimuli, impaired germinal center formation, and reduced numbers of B-cells in the marginal area (14). Furthermore, inhibition of c-Rel by small hairpin RNA results in reduced cell survival and cell cycle progression in murine lymphoma B-cell lines (15). Recently, establishment of a B-cell specific c-Rel overexpressing mouse model demonstrated that c-Rel led to expansion of germinal center B-cells (16). All these data underscore a direct link between c-Rel and B-cells especially those of the germinal center.

Despite its association with REL amplification, it is notorious that GCB-DLBCLs do not exhibit NF-*κ*B signature. This paradox led to question the functional relationship between c-Rel and GCB-DLBCLs. To shed light on the role of c-Rel in these tumors, we analyzed c-Rel DNA-binding activity in DLBCLs together with the gene expression profile of tumors, patient survival, genetic abnormalities, and *REL* imbalances. The role of c-Rel on apoptosis was functionally evaluated *in vitro*.

## Materials and Methods

### Tumors and Patient Cohorts for EMSA Studies, Transcriptomic and Overall Survival Analyses

Clinical data of the patients studied are presented in **Supplementary Tables S1** and **S2** and in **Supplementary Materials and Methods**. Tumors were diagnosed according to the World Health Organization classification (17). The study was performed with approval of an Institutional review board and written informed consent was obtained from all participants at the time of enrollment. All tumor samples were reviewed for tumor infiltration, and selected cases were those for which tumor infiltration was over 90%. EMSA and transcriptomic analysis (HGU133 +2.0 Affymetrix Gene Chip microarray; accession number GSE87371; **Supplementary Table S3**) were performed on frozen material (see **Supplementary Materials and Methods** for details).

Survival and Cox multivariate analysis was done with the Survival package (URL: https://github.com/therneau/survival). Overall survival (OS) was evaluated from the date of enrollment to the date of death from any cause. C-Harrel concordance index was calculated as described (18). Details are given in the **Supplementary Materials and Methods**.

### Real-Time Quantitative Reverse-Transcription PCR

Quantification of REL mRNA was done after extraction of total RNA from frozen biopsies using the TaqMan^®^ Universal PCR Master Mix and TaqMan^®^ Gene Expression Assays: *REL*, Hs00968436_m1; and *HPRT1*, Hs02800695_m1. The *HPRT1* gene was used as a reference gene for the control of amplification (details are in **Supplementary Materials and Methods**).

### Analysis of Gene Copy Number Aberrations; BCL2, BCL6, and MYC Translocations; and Oncogenic-Related Mutations

Gene copy number aberrations were identified by performing Comparative Genomic Hybridization (CGH) on 180 patients after whole-genome amplification, using Agilent SurePrint G3 4 × 180 K microarrays, and analysis was done with GISTIC version 2.0.22 as previously described (19). Genomic imbalance complexity was calculated as the number of all gene loci for which the GISTIC score was −2 (homozygous deletion) or +2 (copy number gain of 2+ copies).

Fluorescence *in situ* hybridization (FISH) for *BCL2*, *BCL6*, and *MYC* translocations was performed as previously detailed (19). Probes are listed in the **Supplementary Materials and Methods**.

The Lymphopanel was designed to identify mutations in 34 important lymphomagenesis genes as detailed previously (19). NGS data was available for 213 patients. Ion Torrent Personal Genome Machine (PGM) Sequencing and PGM data analysis were performed as previously described (19).

### Eukaryote Expression Vectors and Cell Lines

Complementary DNAs (cDNAs) for *REL*, super-repressor I*κ*Bα_S32,36A,12_, I*κ*B*ε*, and Luciferase were cloned into the previously described pRT-1 doxycycline-inducible episomal vector (20). The inducible bidirectional promoter allows concomitant expression of the cDNA of interest and a membrane marker, truncated NGFR (NGFRt). The GCB SUDHL-4 cell line is from the L Staudt group (4). The cell cycle arrested EREB2–5 cell line is described in the **Supplementary Materials and Methods** and has been published (21). Cytoplasm and nuclear extracts and immunoblot were performed as previously described (20). Antibodies and apoptosis experiments are described in the **Supplementary Materials and Methods**.

## Results

### DNA-Binding Activity of c-Rel Was Related to GCB-DLBCLs

**Figure 1:**
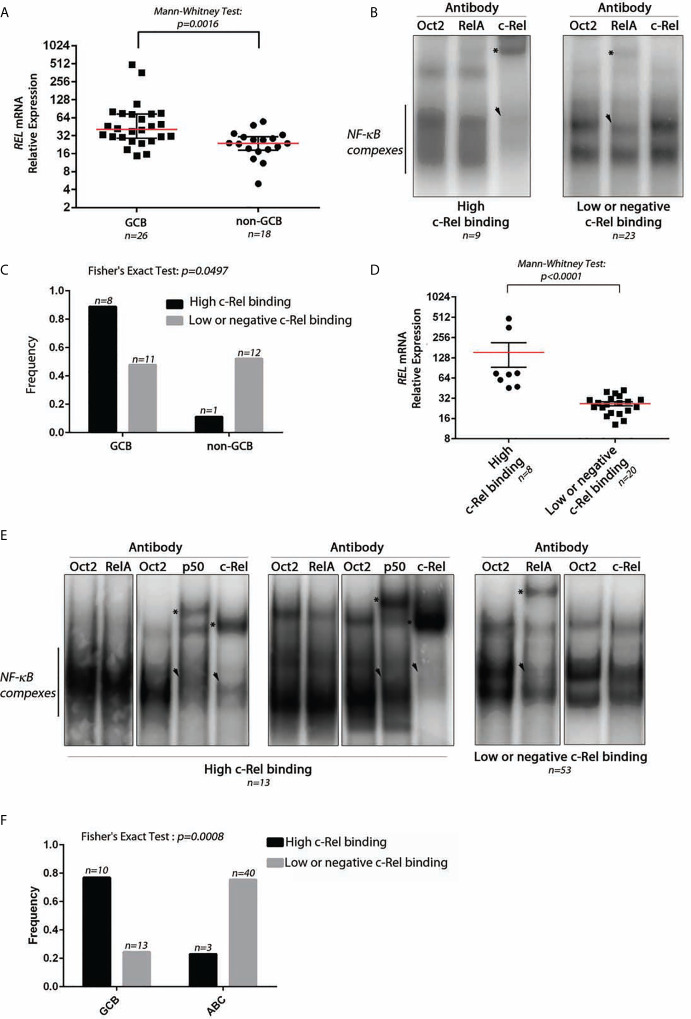
Expression and DNA-binding activity of c-Rel in DLBCLs. **(A)** Relative expression level of *REL* mRNA using TaqMan quantitative PCR from 26 GCB-DLBCL and 18 non-GCB-DLBCL samples from a test series. The red line indicates the median with interquartile range. The approximate *p-value* of a Mann–Whitney test is indicated. **(B)** Two representative EMSAs showing NF-*κ*B complex DNA-binding on a *κ*B binding site containing probe from DLBCL cases with high c-Rel binding (left) and low or negative c-Rel binding (right). *n* indicates the number of cases with the same DNA-binding profile. The use of anti-RelA, anti-c-Rel, or anti-Oct2 (irrelevant control) antibodies is noted at the top. Asterisks point on the super-shifted EMSA signal. Arrowhead indicates decreased NF-*κ*B complex DNA-binding. **(C)** Frequencies of high c-Rel binding and low or negative c-Rel binding among GCB-DLBCLs and non-GCB-DLBCLs. **(D)** Relative expression levels of *REL* mRNA using TaqMan quantitative PCR from eight high c-Rel binding DLBCLs and 20 low or negative c-Rel binding DLBCLs. The red line indicates the median with interquartile range. The approximate *p-value* of a Mann–Whitney test is indicated. **(E)** DNA-binding activity of c-Rel from the training cohort of the GHEDI (Dubois) DLBCL series. EMSAs with supershift experiments (anti-Oct2, RelA, p50, or c-Rel antibodies) were done with proteins extracted from 66 frozen biopsies from 43 ABC-DLBCLs and 23 GCB-DLBCLs. Representative EMSAs showing NF-*κ*B complex DNA-binding on a *κ*B binding site containing probe from DLBCL cases with high c-Rel binding (left) and low or negative c-Rel binding (right). *n* indicates the number of cases with the same DNA-binding profile. Antibodies used for supershift are indicated at the top. Asterisks point on the super-shifted EMSA signal. Arrowhead indicates decreased NF-*κ*B complex DNA-binding. **(F)** Frequencies of high c-Rel binding and low or negative c-Rel binding among GCB-DLBCLs and ABC-DLBCLs.

We studied the *REL* mRNA expression and DNA-binding activity on a test series of 48 DLBCLs classified as GCB (30/48) or non-GCB (18/48) according to the Hans’s algorithm (see **Supplementary Figure S1** for the analysis pipeline). As shown in **Figure 1A**, *REL* mRNA levels tended to be higher in GCB-DLBCLs (Mann–Whitney Test, *p = 0.0016*). EMSA with super-shift was performed to assess the RelA and c-Rel DNA-binding activities for 32 frozen tumor samples (19 GCBs and 13 non-GCBs; **Figure 1B** as a typical result). A high c-Rel DNA-binding activity was defined as a c-Rel DNA-binding higher than that of RelA in gel shift assays. Using this criterion, patients were divided into those with a high and a low or negative c-Rel DNA-binding activity. High c-Rel DNA-binding activity was more frequently found in GCB-DLBCLs when compared to non-GCB subgroup (**Figure 1C**, Fisher’s Exact Test, *p = 0.0497*). Cases with strong c-Rel DNA-binding were those with increased *REL* mRNA expression (**Figure 1D**, Mann–Whitney Test, *p < 0.0001*).

To confirm these results, an independent series of 66 DLBCLs, 43 ABCs, and 23 GCBs classified according the cell of origin and issued from the GHEDI cohort (22) was analyzed. A typical EMSA result with c-Rel DNA-binding-high, low, or negative is shown in **Figure 1E**. Consistently with the test series, the 10/13 (77%) DLBCL cases with high c-Rel binding were GCBs, while most low or negative c-Rel binding cases were ABCs (**Figure 1F**, Fisher’s Exact Test, *p = 0.0008*).

### The c-Rel Signature Is a Hallmark of the Majority of GCB-DLBCLs

**Figure 2:**
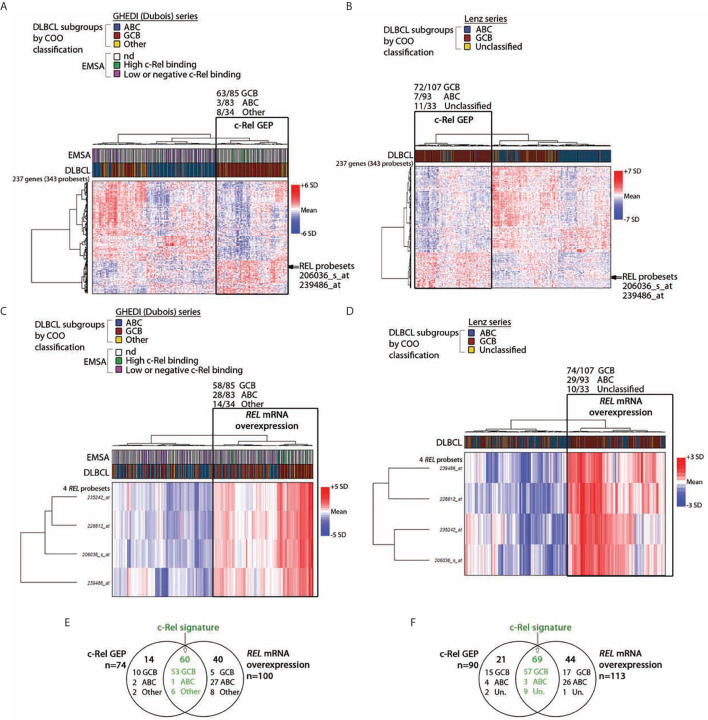
c-Rel related transcriptomic signature of DLBCL patients. By comparing cases with high and low or negative c-Rel DNA-binding, a set of differentially expressed 343 probesets/237 genes with a fold change of two, and a *p-value <0.05* was selected after LIMMA analysis of 66 DLBCLs from the GHEDI cohort for which EMSAs with super-shift were performed. **(A, B)** Unsupervised hierarchical Ward clustering of the whole GHEDI cohort and the Lenz series respectively with the 343 selected probesets. Lines and columns of the heatmap correspond to probesets and patients respectively. Up- and down-regulated genes are color coded according to the color scale shown on the right of the heatmap. The blue, red, and yellow color codes above the heatmap give the original COO ABC/GCB/Other and the ABC/GC/Unclassified diagnosis of the GHEDI and Lenz series respectively. Position of the c-Rel probsesets on gene clusters is indicated by an arrow. For the GHEDI series, high and low c-Rel binding cases by EMSA are color coded in green and purple respectively (nd: EMSA not done). The branch of patients with the c-Rel gene expression profile (c-Rel GEP) is highlighted by a black rectangle on each heatmap. Number of COO cases within this specific branch is indicated at the top of the black rectangle. **(C, D)** Unsupervised hierarchical Ward clustering of the entire GHEDI cohort and the Lenz series respectively with c-Rel probesets. Orientation of the heatmap and color codes are the same as in panel **(A)** The branch of patients overexpressing *REL* mRNA is highlighted by a black rectangle on each heatmap. Number of COO cases within this specific branch is indicated at the top of the black rectangle. **(E, F)** Venn diagram showing the overlap of patients between c-Rel GEP and *REL* mRNA overexpression corresponding to c-Rel signature (in green). Number of cases in each group is indicated. Un., unclassified.

To identify c-Rel DNA-binding activity associated genes, the Affimetrix gene expression profiles of the 13 DLBCL cases of the GHEDI series with a high c-Rel DNA-binding activity were compared to the 53 other cases with a low or negative c-Rel DNA-binding activity (see **Supplementary Figure S2** for the experimental design and the analysis pipeline). With a fold change of two and a Benjamini–Hochberg adjusted *p-value* below 0.05 between high and low or negative c-Rel binding cases, a set of 343 probesets/237 genes was selected after LIMMA analysis (**Supplementary Table S4**). This c-Rel gene expression profile (GEP) was used to clusterize the entire GHEDI series (83 ABCs, 85 GCBs, and 34 Others). PMBLs were excluded from the analysis. As shown in **Figure 2A**, 74/202 (37%) cases exhibited a c-Rel GEP: 63/85 (74%) GCBs, 3/83 (4%) ABCs, and 8/34 (24%) Others. Among the 128 patients with a non-c-Rel GEP 80 (63%), 22 (17%), and 26 (20%) were ABCs, GCBs, and Others respectively (Chi2 Test, *p << 10^−6^*).

Of note, the 10 GCB-DLBCLs with a high c-Rel DNA-binding activity by EMSA were all coclusterized with patients exhibiting the c-Rel GEP. In contrast, 47/53 (89%) EMSA cases with low or negative c-Rel DNA-binding activity were coclusterized with patients that did not exhibit the c-Rel GEP (Fisher’s exact test, *p = 8.10^−6^*). Therefore, GEP and EMSA results were highly concordant, strongly suggesting that c-Rel was transcriptionally active and showing the c-Rel GEP was indeed associated with a majority of GCB-DLBCLs.

To validate this result, the DLBCL series published by Lenz et al. (23) (93 ABCs, 107 GCBs and 33 Unclassified) was also analyzed with these 343 probesets. As shown in **Figure 2B**, this analysis gave similar results since 90/233 (39%) cases were clusterized together with the c-Rel GEP, among them 72/107 (67%) GCBs, 7/93 (8%) ABCs, and 11/33 (33%) Unclassified (Chi^2^ Test, *p << 10^−6^*). Overlaps between genes from clustering of these two independent series were almost perfect, indicating the reproducibility of clustering between both series (**Supplementary Table S5**).

Among the up-regulated genes in GCB-DLBCLs with the c-Rel GEP was the *REL* gene. Restricting the analysis to the four *REL* probesets led to clusterize 100/202 (50%) cases of the GHEDI series, among them 58/85 (68%) GCBs, 28/83 (34%) ABCs, and 14/34 (41%) Others (**Figure 2C**, Chi^2^ Test, *p = 2.5.10^−5^*). Similarly, clustering of the Lenz series with the *REL* probesets identified 113/233 (48%) cases with increased *REL* mRNA expression among them 74/107 (69%) GCBs, 29/93 (31%) ABCs, and 10/33 (30%) Unclassified (**Figure 2D**, Chi^2^ test, *p = 4.5.10^−8^*). These cases were those with *REL* mRNA overexpression. This was in contrast with RELA or RELB because no association was established between mRNA expression of these two NF-*κ*B subunits and the three COO subsets (not shown). To be stringent and because we have experimental evidence showing that over-expression of *REL* mRNA is associated with nuclear translocation of c-Rel protein with increased transcriptional activity (see below), we defined the c-Rel signature by both c-Rel GEP and *REL* mRNA overexpression. Among cases with the c-Rel signature, 53/60 (88%) and 57/69 (83%) were GCBs for the GHEDI and Lenz series respectively (**Figures 2E, F)**.

### Relationships Between c-Rel and ABC-GCB Gene Expression Profiles

In the next step, we investigated the relationship between c-Rel and ABC-GCB gene expression profiles (GEPs). On one hand, we first selected a set of 424 genes (622 probesets) that were differentially expressed with a two-fold change and an adjusted *p-value* below 0.05 between COO classified ABC and GCB-DLBCLs from the same series of 66 cases tested by EMSA. This “re-established” ABC-GCB GEP consisted of 177 (279 probesets) and 247 (343 probesets) up-regulated genes in ABC (ABC-up) and GCB (GCB-up) DLBCLs respectively (**Supplementary Table S6**). It included 18/44 (41%) genes of the ABC/GCB Wright’s predictor (2) such as *FOXP1*, *CCND2*, *IRF4*, *TCF4*, or *IL16* for ABCs and *LMO2*, *MYBL1*, or *VCL* for GCBs.

**Figure 3:**
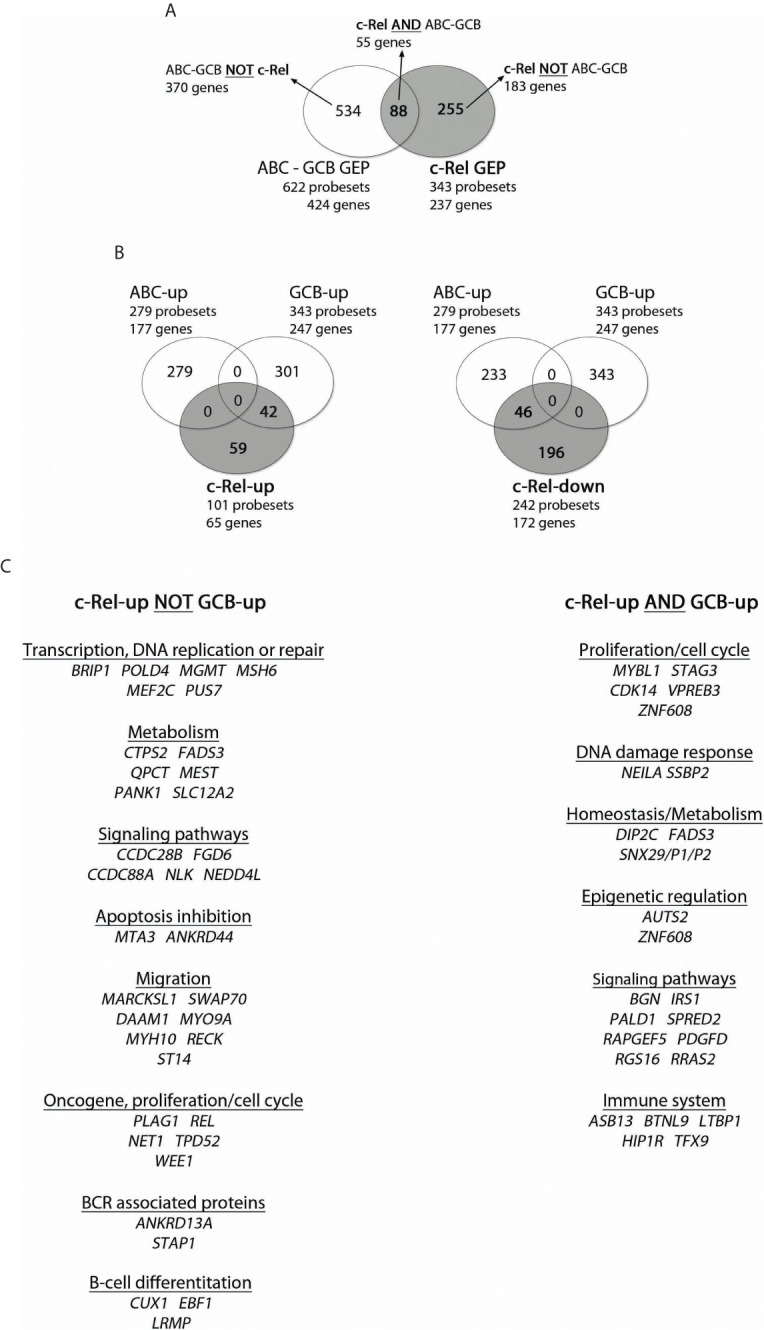
Overlap between c-Rel and ABC-GCB gene expression profiles (GEPs). c-Rel GEP (237 genes, 65 up-regulated genes with 101 probesets, and 172 down-regulated genes with 242 probesets) and ABC-GCB GEP (424 genes, 177 up-regulated genes with 279 probesets, 247 down-regulated with 343 probesets) were established (fold change ≥2) from the 66 DLBCL cases of the GHEDI cohort for which the c-Rel DNA-binding profile was established. **(A)** Venn diagram showing overlaps of probesets and genes between c-Rel GEP and ABC-GCB GEP. Set of genes named “c-Rel AND ABC-GCB” corresponds to the intersection between c-Rel and ABC-GCB GEP with 55 genes and 88 probesets (list in **Supplementary Table S9**). “ABC-GCB NOT c-Rel” are genes found in ABC-GCB GEP but not in c-Rel GEP. “c-Rel NOT ABC-GCB” are genes differentially expressed in c-Rel GEP but not in ABC-GCB GEP. This nomenclature is used in **Figure 5**. **(B)** Venn diagrams showing overlap between c-Rel up-regulated genes (at left) or c-Rel down-regulated genes (at right) and ABC or GCB up genes. Indicated number corresponds to probesets that overlap or not. **(C)** c-Rel-up NOT GCB-up and c-Rel-up AND GCB-up gene lists. Genes are ordered related to their function.

On the other hand and as mentioned above, the c-Rel GEP consisted of 237 genes: 65 up-regulated genes (101 probesets) and 172 down-regulated genes (242 probesets). The intersection between c-Rel and ABC-GCB GEP contained 55 genes (88 probesets) (**Figure 3**, and **Supplementary Table S7**). As shown in **Figure 3**, Venn diagrams indicate that c-Rel up-regulated genes overlapped partially, but only, with the GCB-up genes (26 genes; 42 probesets). Oppositely, c-Rel down-regulated genes overlapped only with ABC-up genes (28 genes; 46 probesets).

This analysis shows that c-Rel GEP only overlapped with GCB GEP, a very strong indication that c-Rel is linked to GCBs and not to ABCs. It also shows that the c-Rel GEP, including *REL* itself, was mainly based on an original set of genes.

### Association of the c-Rel Signature With Genomic Alterations Related to GCB-DLBCLs

**Figure 4:**
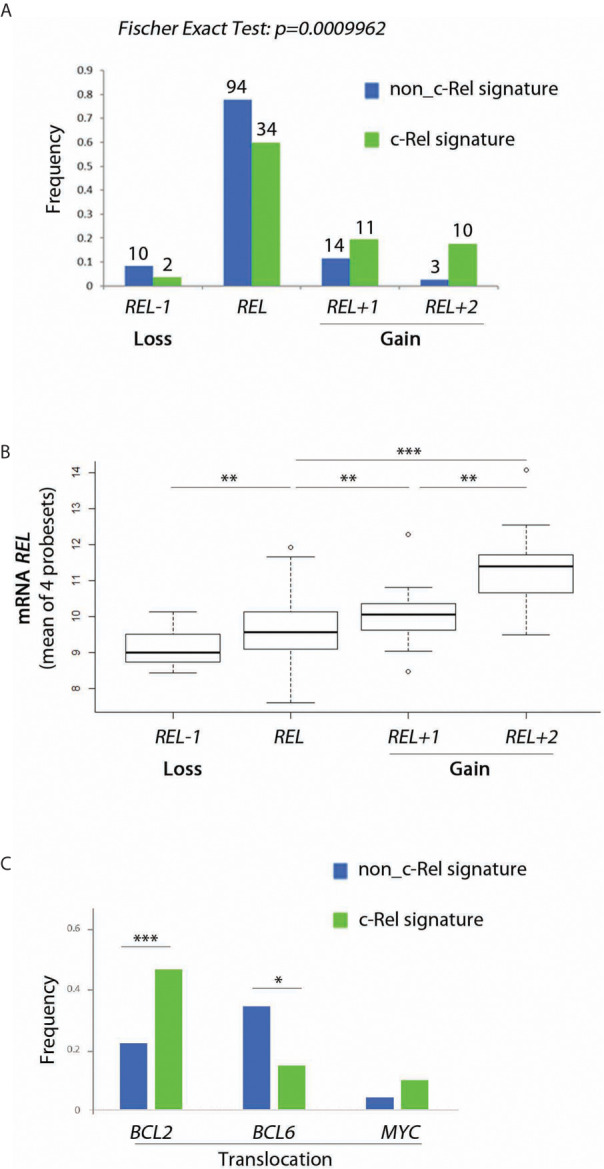
Relationship between c-Rel signature, *REL* gain, *REL* mRNA expression and *BCL2*, *BCL6* and *MYC* translocations for patients from the GHEDI cohort. **(A)** Frequency of *REL* deletion or gain according to c-Rel signature. **(B)** *REL* mRNA expression levels according to *REL* loss or gains. **(C)** Frequency of *BCL2*, *BCL6*, and *MYC* translocations according to the c-Rel signature. In **Figures 3A, C**, green and blue bars indicate tumors respectively expressing or not the c-Rel signature. In **Figures 3A, B**, *REL* deletion, no *REL* number aberrancy, gain of one *REL* copy, and of two or more *REL* copies are encoded by *REL−1*, *REL*, *REL+1* and *REL+2* respectively. Statistical significance was determined by t-test (***p<0.001; **p<0.01; *p<0.05).

The c-Rel signature was associated with increased frequencies of *REL* locus gains (**Figure 4A**, Fisher’s Exact Test, *p = 10^−4^*). Consistently, cases with *REL* gains had higher levels of *REL* mRNA (**Figure 4B**). Frequencies of *BCL2* gene translocation were increased in cases with the c-Rel signature (**Figure 3C**, Fisher’s Exact Test, *p = 1.2.10^−6^*). Conversely, *BCL6* translocation was associated with cases with the non-c-Rel signature, while *MYC* translocation was equally rare in both cases (**Figure 4C**, Fisher’s Exact Test, *p = 0.023* and *p = 0.24* respectively).

**Figure 5:**
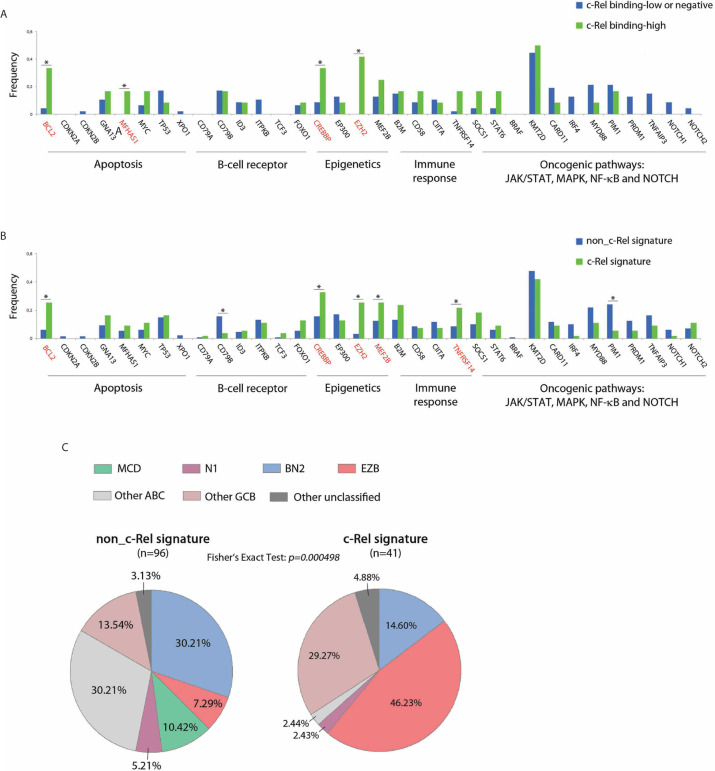
c-Rel related oncogenic mutations in DLBCL patients from the GHEDI cohort. **(A, B)** Mutation frequencies of 66 cases analyzed by EMSA according to c-Rel DNA-binding activity and the whole GHEDI series according to c-Rel expression signature. Genes known to be mutated at high frequencies in GCB DLBCLs are highlighted in red. The functional pathways are indicated at the bottom of the graph. *: Fisher exact test *p-value < 0.05*. **(C)** Percentage of 134 DLBCL cases that could be reclassified in BN2 (based on *BCL6* fusions and *NOTCH2* mutations), EZB (based on *EZH2* mutations and *BCL2* translocations), MCD (based on *MYD88L265P* and *CD79B*), N1 (based on *NOTCH1* mutations), “other ABC”, “other GCB”, and “other unclassified” subtypes according to the c-Rel expression signature.

Mutational status of 34 genes recurrently altered in DLBCLs was explored in 183/202 cases from the GHEDI cohort, including 59/66 (89%) cases for which c-Rel DNA-binding activity was assessed. As shown in **Figures 5A, B**, mutational profiles of cases with high c-Rel DNA-binding or with the c-Rel signature were closed, being characterized by increased frequencies of *BCL2*, *CREBBP*, *EZH2*, *MEF2B*, and *TNFRSF14* mutations and decreased frequency of *CD79B* and *PIM1* mutations when compared to cases with the non-c-Rel signature. FISH cytogenetics and mutational status of *MYD88*, *CD79B*, *NOTCH1*, *NOTCH2, BCL2*, and *EZH2* classified 137/202 (67%) DLBCL cases from the GHEDI cohort according to genetic subtypes defined by Schmitz et al. (5). We found 35 BN2 (based on *BCL6* fusions and *NOTCH2* mutations), 26 EZB (based on EZH2 mutations and BCL2 translocations), 10 MCD (based on the co-occurrence of MYD88L265P and CD79B mutations), six N1 (based on NOTCH1 mutations), 30 “other ABC”, 25 “other GCB”, and five “other unclassified” cases (**Supplementary Table S8**). As shown in **Figure 5C**, the c-Rel signature was mostly associated with the GCB EZB subtype, while this signature was absent in MCD cases and almost absent from the “other ABC” subtype. Of note, survival analysis indicated that, even if numbers were small, c-Rel signature tended to be associated with good prognosis among EZB cases (**Supplementary Figure S3**).

Thus, the c-Rel signature was related to *REL* locus gains and to other mutations known to be associated with GCB-DLBCL cases such as *BCL2* translocation and *EZH2* mutations. Consequently, the c-Rel signature subtype was part of the EZB DLBCL subtype and had a favorable impact on patient survival.

### The c-Rel Signature Is Associated With Decreased Genomic Complexity, Specific Gene Imbalances and Better Overall Survival

**Figure 6:**
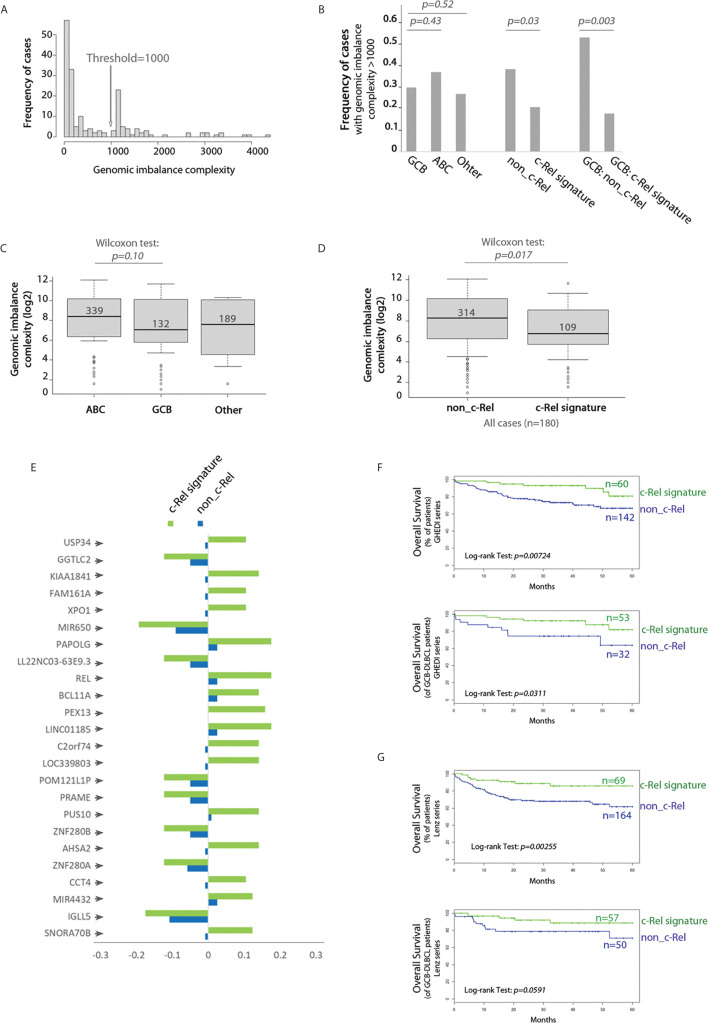
c-Rel related gene copy number aberrations and overall survival. **(A)** Frequency histogram of the genomic imbalance burden reveals a bimodal distribution with a threshold at 1,000. **(B)** Frequency of DBCL cases with a genomic imbalance burden >1,000 according to the COO classification (left), absence (non_c-Rel) or presence (c-Rel signature) of the c-Rel signature (middle and right) among the entire GHEDI series (left and middle) or GCB-DLBCLs only (right). Chi^2^ test p-values are indicated on the top. **(C)** Boxplot of the genomic imbalance burden according to the COO classification. **(D)** Boxplot of the genomic imbalance burden according to the absence (non-c-Rel) or presence (c-Rel signature) of the c-Rel signature. **(C, D)** Median number of gene imbalances is indicated by the black solid line. Wilcoxon test p-values are shown on the top. **(E)** Frequency of cases with gene copy number aberrations for the 24 gene loci specifically imbalanced in DLBCL cases with the c-Rel signature. Negative values mean deletion while positive values mean amplification. **(F, G)** Kaplan–Meier overall survival curves of all (upper panel) or GCB (lower panel) patients from GHEDI and Lenz series respectively according to expression of the c-Rel signature (c-Rel signature, green curve) or not (non-c-Rel, blue curve). Number of cases is indicated on the right. The log-rank p-value is given within each graph.

We next analyzed the whole genome imbalances of the GHEDI series by CGH-array. With the GISTIC pipeline, each of the 23,636 gene loci was scored between −2, −1, 0, 1, 2 according to the level of gene loss (−2 meaning loss of both alleles and +2 meaning gain of two copies or more). Because of the uncertainties of CGH-array signal quantification, only gene scores of −2 or +2 were taken into account. With these criteria, the genomic imbalance complexity was estimated as the number of all genes for which loci were deleted or amplified. Histogram frequencies of the number of gene loci imbalances revealed a bi-modal distribution with a threshold at 1,000 (**Figure 6A**). The proportion of cases with a genomic imbalance complexity over this threshold was increased in ABC-DLBCLs when compared to GCB or “Other” DLBCLs but without reaching statistical significance (**Figure 6B**). By contrast, the c-Rel signature was associated with a marked decrease in cases with a genomic imbalance complexity above 1,000 (**Figure 6B**, Wilcoxon test, *p = 0.017*). This was still true and even more striking when this analysis was restricted to GCB cases only (**Figure 6B**, Wilcoxon test, *p = 0.003*). Indeed while being not significantly different between ABC and GCB-DLBCLs, the genomic imbalance complexity of cases with the c-Rel signature was significantly decreased when compared to non-c-Rel cases (**Figures 6C, D**).

To search for specifically altered gene loci in c-Rel cases, we used as a criterion that a gene copy number aberration had to be retrieved in at least 10% of c-Rel cases with a 50% frequency increase when compared to non-c-Rel cases. With this condition a list of 24 genes was selected (**Figure 6E** and **Supplementary Table S9**). All these genes, including *REL*, were located in the 2p15-16.1 chromosome region when amplified and in the 22q11.22 chromosome region when deleted (**Supplementary Table S9**). Amplification of 2p15-16.1 chromosome region could be expected (24) and is associated with transformation from follicular lymphoma to DLBCL (25). Deletion of the 22q region was reported once and could be associated in DLBCLs with a more advanced clinical stage (26). Among these genes, some are already known in DLBCLs. For example, we found that *USP34* (*Ubiquitin Specific Peptidase 34*) and *BCL11A* (*BAF Chromatin Remodeling Complex Subunit BCL11A*) were co-amplified with *REL* itself. Co-amplification of these three gene loci is found in transformed DLBCLs and indicates a gain of chromosome 2p15-16.1 (25). Amplification of the *XPO1* gene (*Exportin 1*, also located on chromosome 2p15) was found in 10.5% c-Rel cases of the GHEDI series. *XPO1* gene mutations have been associated with PMBLs (22). High XPO1 expression has been reported in DLBCL and its specific inhibition is targetable by the selinexor agent (27). *PRAME* (*Preferentially Expressed Antigen In Melanoma*, located in 22q11.22) gene, which overexpression is associated with a worse prognosis in DLBCLs (28), was deleted in 12.3% c-Rel cases, suggesting these cases were oppositely associated with expression of this gene. Deletion of *IGLL5* (*Immunoglobulin Lambda Like Polypeptide 5*, also located in 22q11.22) locus, which is one of the most mutated gene in DLBCLs, was found in 17.5% c-Rel cases.

Since genomic imbalance complexity was decreased in DLBCL cases with the c-Rel signature and that these cases exhibited specific gene copy number aberrations that could be associated with prognostic, we examined the overall survival (OS) impact of the c-Rel signature. DLBCL cases from both GHEDI and Lenz series with the c-Rel signature exhibited an increased overall survival (OS) (upper panel of **Figures 6F, G**). Interestingly, and most likely related to the lower genomic imbalance burden, this better OS tended to be confirmed on GCB cases (lower panel of **Figures 6F, G**).

### Overexpression of c-Rel Was Associated With Increased Protection Against Apoptosis

**Figure 7:**
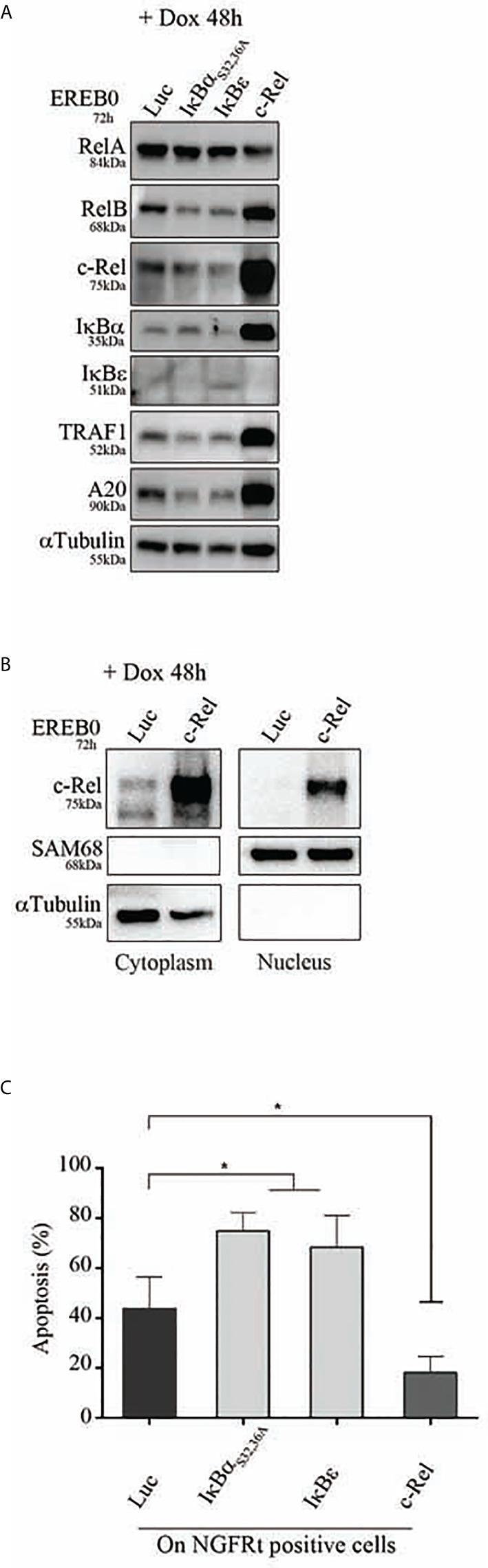
Apoptosis of c-Rel overexpressed human B-cells. EREB2-5 cells were stably transfected with the doxycycline-regulatable pRT-1 vector coding for Luciferase (Luc), I*κ*B*α*_S32,36A_, I*κ*B*ε*, or c-Rel. After estradiol starvation for 24 h (EREB0), cells were treated with doxycycline (+ Dox) for 48 h. **(A)** Immunoblots detecting the expression of RelA, RelB, c-Rel, I*κ*B*α*, I*κ*B*ε*, TRAF1, A20 as well as *α*Tubulin as loading control. **(B)** Detection of cytoplasmic and nuclear c-Rel protein by immunoblot. SAM68 and *α*Tubulin revelations are nuclear and cytoplasmic markers, respectively. **(C)** Percentages of Annexin-V-positive cells gated on NGFRt positive cells. Statistically significant difference (Mann–Whitney test) is indicated by *(*p ≤ 0.05*).

To assess the *in vitro* functional role of c-Rel on proliferation and protection against apoptosis, we used the cell cycle arrested EREB2.5 cell line. This cell line, which proliferation can be arrested by estradiol withdrawal, allows assaying the role of any putative oncogene without interference with its proliferation potential. Cells were stably transfected with a doxycycline regulatable episomal vector allowing for expression of c-Rel. In B-cells, c-Rel is regulated by I*κ*B*α* and I*κ*B*ε* inhibitors with a predominant role of the latter (29). In consequence and in addition to the luciferase control, we also transfected cells to over-express the I*κ*B*α*_S32,36A_ super-repressor (20) and the I*κ*B*ε* inhibitor. As shown in **Figure 7A**, c-Rel overexpression in estradiol starved EREB2.5 B-cells (resting state) was associated with increased expression of RelB, I*κ*B*α*, TRAF1, and A20 proteins (known to be NF-*κ*B targets) when compared to luciferase-expressing cells, suggesting a constitutive NF-*κ*B transcriptional activity. Indeed, c-Rel was constitutively found in the nucleus of cells when over-expressed (**Figure 7B**). No effect of c-Rel was seen on cell proliferation in this model (not shown). While induction of I*κ*B*α* and I*κ*B*ε* inhibitors increased cell apoptosis, c-Rel protected resting EREB2.5 B-cells against cell death when compared to luciferase control cells (**Figure 7C**). Reciprocally, inhibition of c-Rel expression in the GCB SUDHL-4 cell line tended to increase cell death (**Supplementary Figure S4**).

Therefore, overexpression of c-Rel was associated with its constitutive nuclear translocation, and protection against cell death.

## Discussion

Here, we describe a new c-Rel transcriptomic signature related to c-Rel DNA-binding activity in GCB-DLBCLs. Results indicate that this new c-Rel signature defined a good prognosis compared to non-c-Rel cases. Cases with the c-Rel signature were more frequently associated with *REL* locus gains, *BCL2* translocation, and *EZH2* mutations. Functionally, overexpression of c-Rel protein was sufficient to induce its nuclear translocation and to protect cells against apoptosis.

The relationship between *REL* gains and R*EL* mRNA overexpression has been previously reported by others (8). Confirming those published by Li et al. (30), our results show that *REL* mRNA levels were higher in GCB than in ABC-DLBCLs. Probably due to immunostaining technical limitations, it is not clear from the literature whether a clear relationship between c-Rel protein expression levels, its nuclear localization and *REL* amplification exists in ABC and GCB-DLBCLs (30, 31). Pham et al. reported significant higher c-Rel DNA-binding activity in GCB compared to ABC-DLBCL subtypes from 14 DLBCL cell lines and 14 DLBCL patient samples using an ELISA-based approach (32). By EMSA with a supershift approach, we show that high c-Rel DNA-binding was more frequently associated with GCB than ABC-DLBCLs in two independent DLBCL series. We also found that *REL* mRNA expression levels were correlated to its c-Rel DNA-binding activity, to c-Rel GEP, and to *REL* amplification. This suggests that *REL* mRNA overexpression is probably a good indicator of c-Rel transcriptional activity. This conducted us to define the c-Rel signature as the association of both *REL* mRNA over-expression and c-Rel GEP. These DLBCL cases were those with the best overall survival in both the GHEDI and Lenz series.

The c-Rel GEP consisted in 237 differentially expressed genes between high c-Rel binding samples and the others. Among up-regulated genes of the c-Rel GEP were *REL* and *NLK* (*Nemo-Like Kinase*). *NLK* has been shown to negatively regulate RelA NF-*κ*B activation by targeting CBP300 or IKK*β* (33). Increased expression of NLK has been found in various solid cancers such as those of the colon or lung (34). The c-Rel GEP also includes *EBF1* or *SWAP70* genes. *EBF1* coding for a B-cell specific transcription factor is involved in immunoglobulin heavy chain translocations in DLBCLs (35), and SWAP70 is known to be associated with B-cell activation, immunoglobulin class switching, migration and homing processes essential for B-cell entry into lymph nodes, and germinal center formation (36). Genes involved in metabolism such as pyrimidine nucleotide or lipid metabolic processes with *CTPS2* (*CTP synthesis from UTP*) and *FADS3* (*fatty acid desaturase 3*) genes were also among the up-regulated genes of the c-Rel GEP. Some of the c-Rel-up and GCB-up subset of genes are involved in cell proliferation, G2/M transition of the mitotic cell cycle, or in DNA repair such as *STAG3*, *CDK14*, and *NEIL1*. Other genes are also regulators of the Ras signaling pathway such as *RAPGEF5*, *RGS16*, and *RRAS2* genes. NF-*κ*B activity in cancer can arise from mutations in upstream regulators such as the Ras pathway (37). Oncogenic KRAS driven cancers require NF-*κ*B anti-apoptotic signals involving c-Rel (38). Furthermore, microRNA dysregulation can target the Ras signaling pathway in germinal center derived lymphomas such as DLBCLs and Burkitt’s lymphomas (39). The *VPREB3* gene, also belonging to this gene subset, has been shown to be over-expressed in germinal center B-cells as well as in DLBCLs with *MYC* amplification (40). Consistent with the previously described importance of c-Rel in germinal center B-cells, various genes that are in the c-Rel-down and ABC-up subset are known to be down-regulated in the germinal center B-cells such as *BATF*, *RAB29*, *CCND2*, *HCK*, *CD44*, and *STAT3*.

Interestingly, cases from the GHEDI series with high c-Rel DNA-binding and c-Rel signature more frequently exhibited the genetic events previously ascribed to GCB-DLBCLs and follicular lymphomas (FL) (1), including *BCL2* translocation, *MEF2B* and *EZH2* gain of function mutations as well as loss of function of the tumor suppressors *CREBBP*, and *TNFRSF14*. MEF2B may control BCL6 which in turn regulates LMO2 and MYBL1 (41), the latter being up-regulated in both GCB and c-Rel signatures. As demonstrated by LM Staudt’s group, the ABC/GCB-DLBCL subgroups can be separated into distinct genetic subtypes, among them EZB, based on *EZH2* mutations and *BCL2* translocation. This subtype mainly corresponds to the GCB-DLBCL cases (5). EZB is also related to *REL* amplification (5). Here, EZB DLBCLs frequently exhibited the c-Rel signature that could suggest a relationship between c-Rel transcriptomic activity and EZH2 dysregulation. This could probably, at least partially, be due to the transcriptional regulation of *EZH2* by c-Rel as previously shown in activated B and T lymphocytes (42). Patients with EZB tumors have a trend toward increased frequency of *MYC* translocation and inferior overall survival when compared to “other GCB” patients (5, 43). The number of cases from the GHEDI series with *MYC* translocation was too low to draw any conclusions. Nevertheless, among all GCB as well as the EZB classified GCB cases, patients with the c-Rel signature had a much more favorable outcome than non-c-Rel patients. This probably illustrates the importance of c-Rel in the biology of GCB cases.

Under normal conditions, c-Rel is expressed in mature B-cells. Its expression is up-regulated throughout B-cell differentiation upon activation mediated by the B-cell receptor (BCR), and CD40 during T-cell contact. Toll-like receptors 4 and 9 also mediate c-Rel expression in B-cells (44). Germinal center formation and maintenance require c-Rel expression in order for B-cells to shuttle between dark and light zones (45). Furthermore, BCR activated B-cells have elevated levels of apoptosis in the absence of c-Rel (46) suggesting that c-Rel provides an important survival signal to reconfigure the BCR in germinal centers. In pathological conditions, we speculate that aberrant increased c-Rel expression and/or activity corresponding to the c-Rel signature may promote tumorigenesis in DLBCL-precursor cells by disturbing the normal dynamics of germinal center B-cell development. In other words, c-Rel could send a survival signal to a germinal center B-cell normally destined to die (after, for example, loss of antigen recognition ability). Like NF-*κ*B continuous activation in MALT lymphoma that is first due to the B-cell immune environment before acquiring its independence thanks to translocations involving *MALT1* and *BCL10* genes, it can be hypothesized that the germinal center environment of the initial tumor cell was first responsible for the c-Rel up-regulation. c-Rel up-regulation would become constitutive over time thanks to acquired genetic events such as *REL* amplification for example or by others not known yet mechanisms including epigenetic, transcriptomic, and/or post-transcriptional oncogenic events. As mentioned above, the dysregulation of the RAS pathway, that we found associated with the c-Rel signature, could be one of this additional oncogenic mechanisms (38). Finally, our *in vitro* results suggest that *REL* mRNA overexpression is associated with nuclear translocation of a transcriptionally active c-Rel protein in the absence of any activation signal, and increased protection against apoptosis in a model of resting human B-cells. We previously showed that overexpressed RelA remains stored in the cytoplasm in the absence of adequate activation signals in the EREB2.5 EBV transformed B-cell model [(20) and unpublished results]. In contrast to RelA, c-Rel does not have a nuclear export signal explaining why *de novo* c-Rel gene transcription and translation can lead to long-term maintenance of nuclear c-Rel-containing heterodimers (47). These different properties of the NF-*κ*B subunits would explain why tumors dependent upon RelA activation must absolutely acquire mutations in the NF-*κ*B activation track while c-Rel overexpression, due for example to *REL* locus gains, would not require additional activation events to achieve c-Rel effects.

The hypothesis that, to exert its oncogenic effects, c-Rel would not need additional genetic events is fully in agreement with the fact that the genomic imbalance complexity of DLBCL cases with the c-Rel signature was markedly decreased. It has been shown that, rather than the antigenic pressure, oncogenic events are the main driving forces for relapse in DLBCLs (48). Like in chronic lymphocytic leukemia in which the cytogenetic complexity is associated with resistance to therapies, increased imbalance genomic complexity in non-c-Rel cases is very likely to be related to genetic instability that would favor the selection of a therapy resistant DLBCL subclone. Oppositely around, the decreased genomic imbalance complexity of c-Rel DLBCL cases would be associated with a better prognosis.

While ABC-DLBCLs are very likely to be associated with RelA activation, the place of c-Rel in DLBCLs remains rather enigmatic since it is associated with GCB-DLBCLs that are not supposed to harbor any NF-*κ*B activity. An explanation for such a discrepancy between the absence of NF-*κ*B signature and *REL* amplification may have been provided by TJ de Jesùs and P Ramakrishnan (49). Indeed these authors showed that c-Rel represses TNF-*α*-induced RelA-mediated transcription. This suggests that long term over-expression of c-Rel could extinguish the RelA signature. In that context, a c-Rel signature would almost never overlap with that of RelA. Consistently, we did not find any overlap in between c-Rel and ABC-DLBCL signatures.

For the first time, we show that a strong c-Rel DNA binding activity was a feature of GCB-DLBCLs. These GCBs specifically exhibited a c-Rel transcriptomic signature, *REL* gains, *BCL2* translocation, *EZH2* mutations, and a favorable prognosis. They also exhibited a decreased genomic imbalance burden and some specific gene copy aberrations that are linked to 2p15-16.1 amplification and 22q11.22 deletion. These could likely be associated with the outcome such as *PRAME* deletion or *XPO1* amplification, the latter being also targetable by selinexor. Functionally, c-Rel was responsible for protection against apoptosis. Thus, c-Rel could very likely participate in cell transformation in a significant proportion of GCB-DLBCLs. As a future direction, the better survival advantage for DLBCL patients with a c-Rel signature could be examined with regard to both sensitivity toward apoptosis under classical therapeutic molecules and the ability of tumor cells to be eliminated by the immune microenvironment. Even if this has to be confirmed on prospective studies, R-CHOP regiment is likely to give very good results on DLBCL tumors with the c-Rel signature. This also raises the question whether the c-Rel signature could become a decision criterion to better choose patients who would very likely be able to benefit from R-CHOP regimen or not. In other words, it would be of importance to find alternative therapeutics for patients whose DLBCL tumors do not exhibit the c-Rel signature and have increased genomic imbalance complexity.

## Data Availability Statement

The datasets presented in this study can be found in online repositories. The names of the repository/repositories and accession number(s) can be found in the article/**Supplementary Material**.

## Ethics Statement

The studies involving human participants were reviewed and approved by the LYSA group Institutional Review Board. The patients/participants provided their written informed consent to participate in this study.

## Author Contributions

NF, VB, and JF designed the study and supervised the work. NF and JF wrote the manuscript. NF, OT, DC, and LP made the experiments. KL, JF, and TM reviewed and selected the cases from the GHEDI series of patients. NF, JF, BP, M-PG, DB, and DT reviewed and selected the cases issued from the CHU of Limoges. JF and J-PJ made the bioinformatics and the statistics. CC-B made the cytogenetics. All authors contributed to the article and approved the submitted version.

## Funding

This study was funded by grants from the Institut National du Cancer (INCA). The group of JF is supported by grants from the Ligue Nationale Contre le Cancer (Equipe labellisée Ligue), the Comité Orientation Recherche Cancer (CORC) and the Haute-Vienne and Corrèze committees of the Ligue Nationale Contre le Cancer. The group of VB is supported by grants from the Institut National du Cancer, Agence Nationale pour la Recherche, the European Union’s Horizon 2020 research and innovation programme under the Marie Skłodowska-Curie grant agreement No 766214, Université de Paris, and Fondation ARC pour la Recherche sur le Cancer (Equipe labelisée), and doctoral funding from IDEX doctoral international program and Societé Française d’Hémathologie (to DC).

## Conflict of Interest

The authors declare that the research was conducted in the absence of any commercial or financial relationships that could be construed as a potential conflict of interest.
