# Supplementary figures and images for: c-Rel Is the Pivotal NF-κB Subunit in Germinal Center Diffuse Large B-Cell Lymphoma: A LYSA Study
Source: Front Oncol. 2021 Apr 20;11:638897. doi: 10.3389/fonc.2021.638897 (PMC8095348; doi:10.3389/fonc.2021.638897)

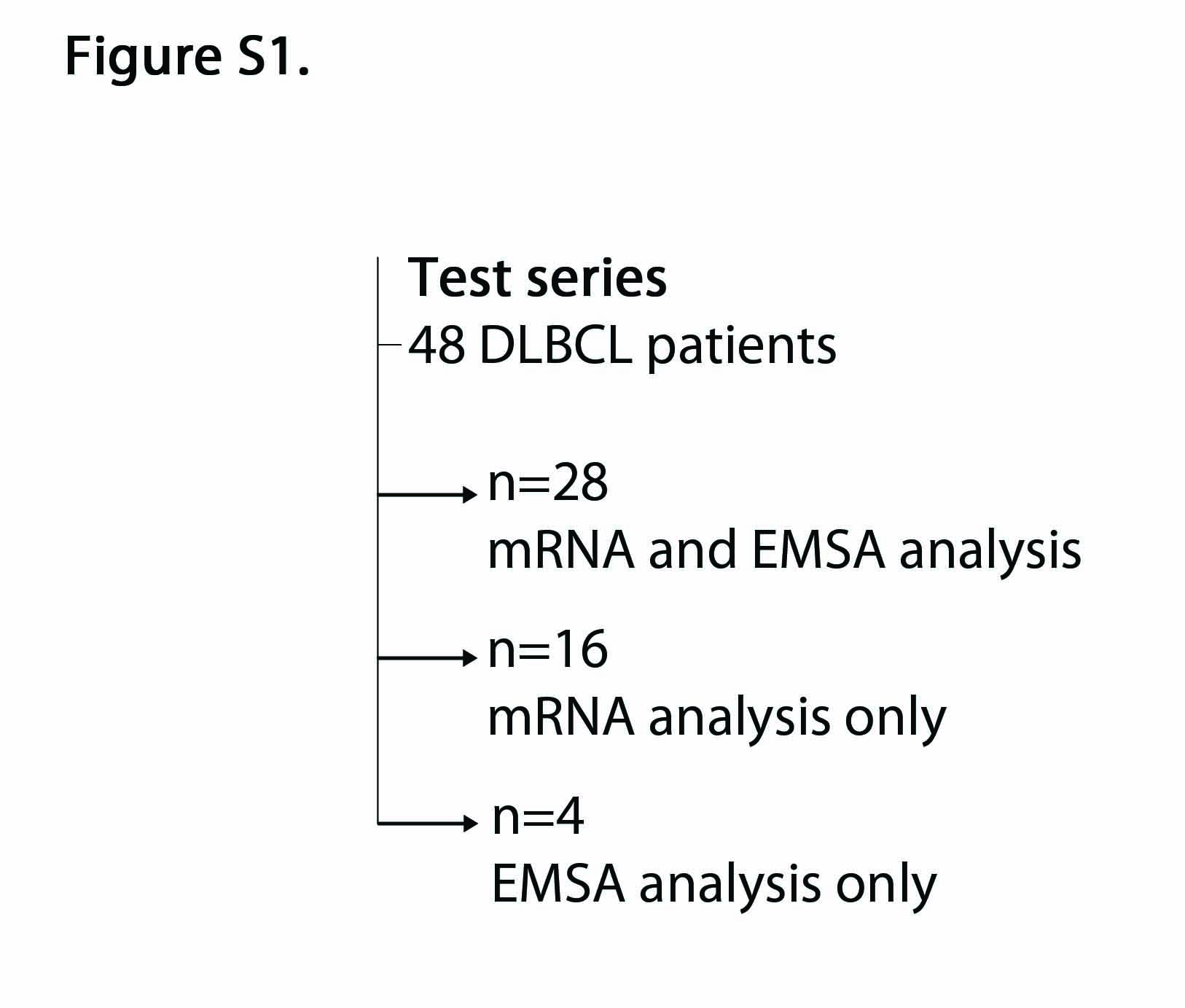

Supplement: Supplementary file 1 [file Image_1.jpeg]

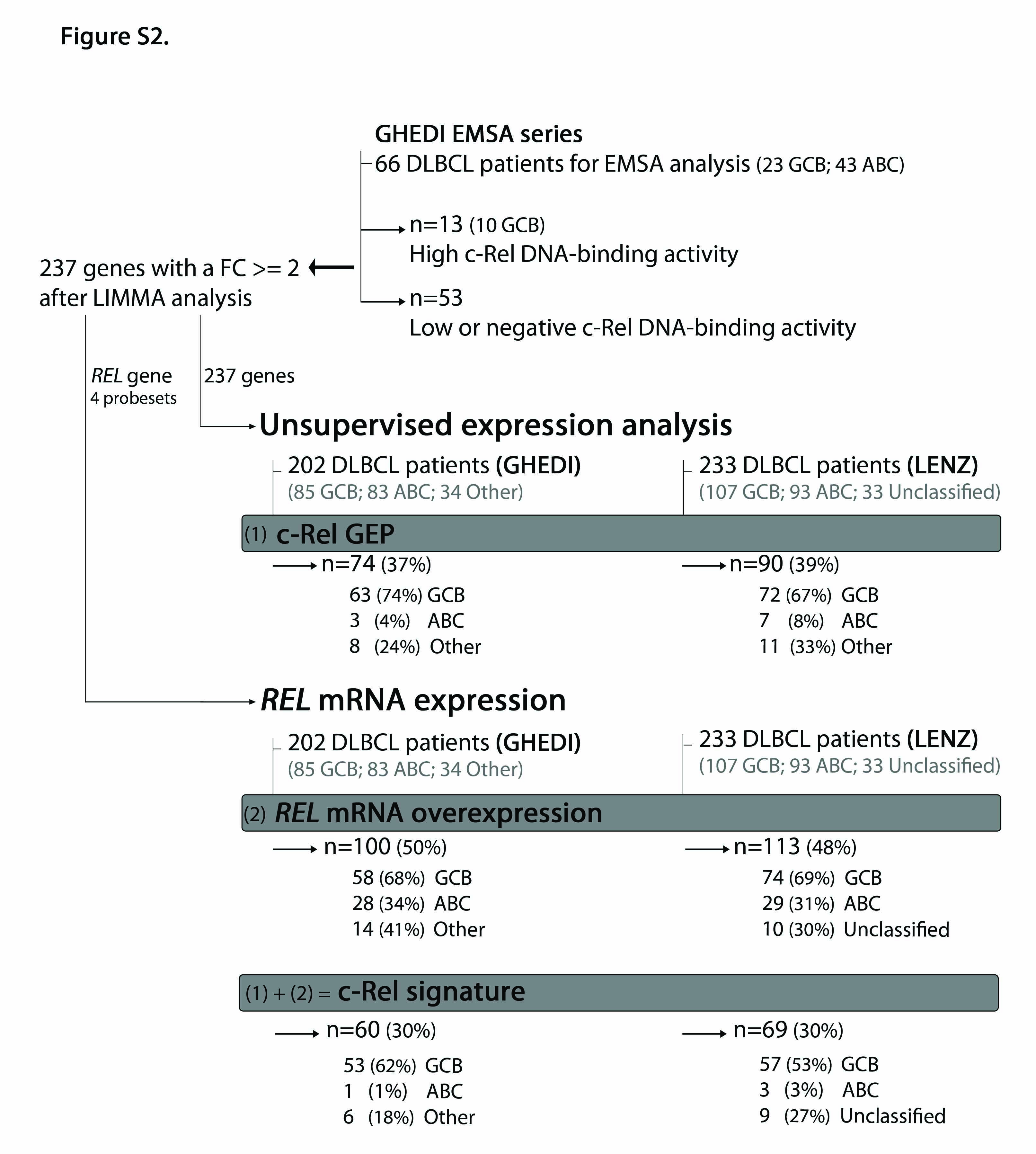

Supplement: Supplementary file 2 [file Image_2.jpeg]

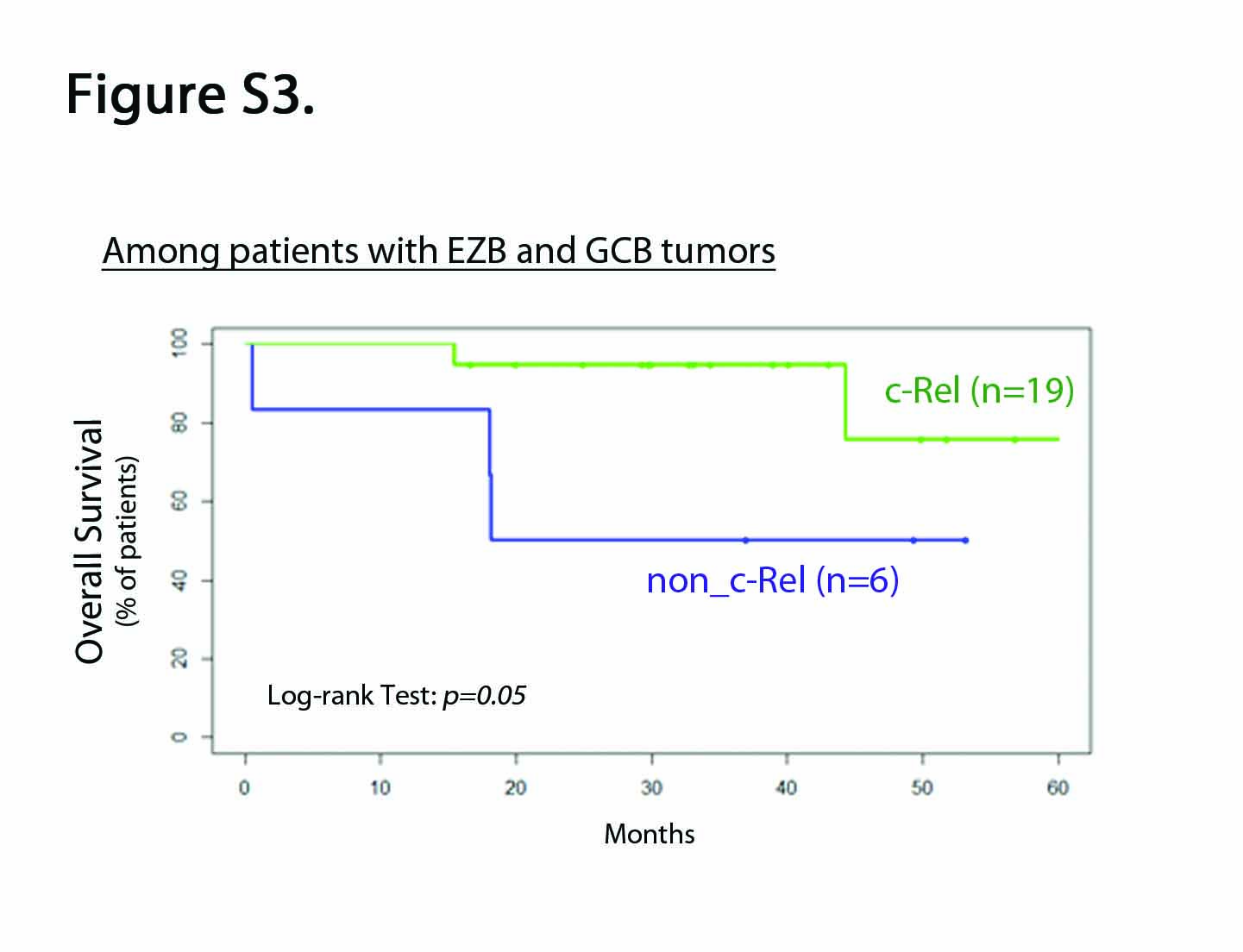

Supplement: Supplementary file 3 [file Image_3.jpeg]

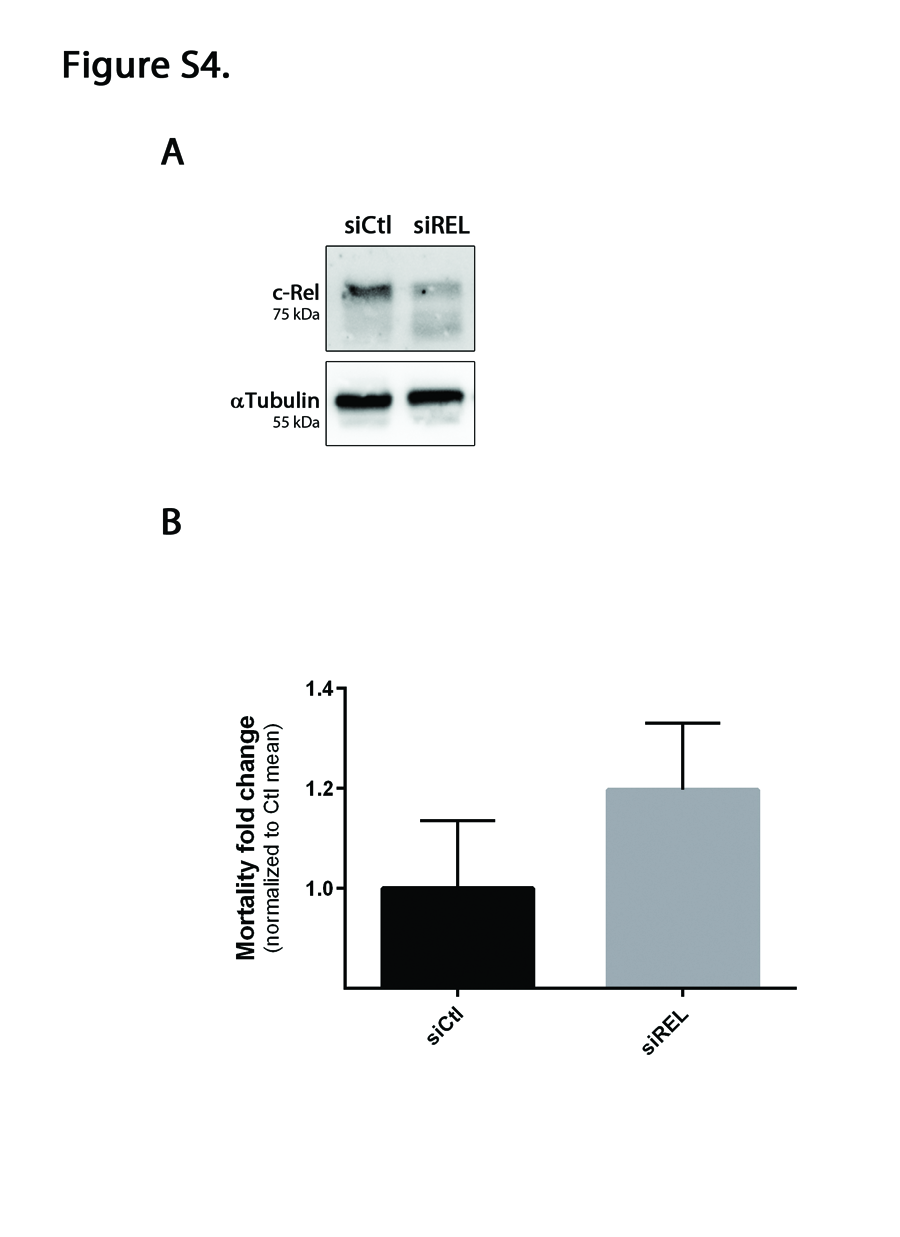

Supplement: Supplementary file 4 [file Image_4.tif]
