# Supplementary material for: c-Rel Is the Pivotal NF-κB Subunit in Germinal Center Diffuse Large B-Cell Lymphoma: A LYSA Study
Source: Front Oncol. 2021 Apr 20;11:638897. doi: 10.3389/fonc.2021.638897 (PMC8095348; doi:10.3389/fonc.2021.638897)
Supplement: Supplementary file 5 [file DataSheet_1.zip › Supplementary Methods.DOCX]

**Supplementary Materials and Methods**

*Tumors and Patient Cohorts and EMSA studies*

For EMSA, the test set comprised a series of 48 tumor frozen samples from DLBCL patients. Clinical data were previously published and summarized in Supplementary Table S1 (1). DLBCL cases were classified as GCB or non-GCB according to the Hans algorithm based on CD10, Bcl-6, and MUM1 expression (2).

Tumor frozen samples of the validation set (n=66) were part (n=220) from a previously published series of DLBCL patients from the GHEDI (*Deciphering the Genetic Heterogeneity of Diffuse large B-cell lymphoma in the rituximab era*) study program including the LNH 98.5 and LNH 2003 LYSA clinical trials with available frozen tumor samples, centralized histopathologic review, and adequate DNA/RNA quality (3). Clinical features of the patients were indicated in Supplementary Table S2.

All DLBCL patients included in this study received standard chemotherapy (CHOP) with anti-CD20 immunotherapy in most cases (Supplementary Tables S1 and S2).

Protein was extracted from frozen tumor sections (30 µm), in 20 mM HEPES pH 7.9, 25% Glycerol, 0.42 M NaCl, 1.5 mM MgCl2, 0.2 mM EDTA, 0.5 mM PMSF, and 10 mM DTT. After ultracentrifugation at 40 000 g, protein extract was used for EMSA. Methods for EMSA are described elsewhere (4).

*Transcriptomic, and Survival Analysis*

For the GHEDI (Dubois) series, the cell-of-origin (COO) molecular classiﬁcation (ABC, GCB, and Other) was based on the gene expression profile established with HGU133 +2.0 Affymetrix Gene Chip arrays (Affymetrix, Santa Clara, CA) and was done using the Bayesian predictor described by Wright *et al.* and adapted by Monti *et al.* (5,6). The HGU133 +2.0 Affymetrix Gene Chip microarray data set has been deposited in the National Center for Biotechnology Information’s Gene Expression Omnibus (https://www.ncbi.nlm.nih.gov/geo; accession number GSE87371) (3). LIMMA analysis was performed on the 12 959 most variable genes (Supplementary Table S3). Unsupervised clustering was done using the LPS package (<https://bioinformatics.ovsa.fr/LPS>) and as previously described (7)). Effect of c-Rel signature and COO classification as covariates on treatment-free survival (TFS) was examined using the Cox proportional hazard model and the c-Harrel concordance index were determined as previously described (8). Briefly, c-Harrel index is designed to estimate the concordance probability (hereafter the informativeness) by comparing the rankings of two independent pairs of survival times Ti,Tj for patients i and j, and prediction the variable xi, xj25. Here xi and xj take the value 0 or 1 according to the criteria studied (ABC vs GCB classification for exemple). The c-Harrel concordance index can also be interpreted as a summary measure of the area(s) under the time dependent ROC curve(s). With each variable being coded as 1 (present) or 0 (absent), the c-Harrel concordance index was calculated with the “rcorr.cens” command of the Hmisc package (<https://github.com/harrelfe/Hmisc>).

*Real-time Quantitative Reverse–Transcription PCR*

Total RNA was extracted from frozen biopsies. Complementary DNAs (cDNAs) were reverse transcribed from 1 µg of total RNA samples using the High Capacity cDNA Archive Kit (Life Technologies, Carlsbad, USA). PCR products were amplified from each cDNA using the TaqMan® Universal PCR Master Mix and TaqMan® Gene Expression Assays: *REL*, Hs00968436_m1; and *HPRT1*, Hs02800695_m1. The *HPRT1* gene was used as a reference gene for the control of amplification. The calculated relative gene expression level was equal to 2^−DDCT^, where DDCT is the delta delta cycle threshold (Ct). Gene expression fold changes were calculated as the ratio of the patient to the control EREB2-5 EBV-immortalized human B-cell line.

*Probes for FISH analysis*

Analysis of *BCL2*, *BCL6*, and *MYC* translocations by FISH was performed using break-apart FISH DNA probes for BCL2/18q21, BCL6/3q27 (probes Y5407, and Y5408; Dako A/S), MYC FISH DNA probe Split Signal (Y5410, Dako A/S) and Vysis LSI MYC dual color, BA rearrangement probe (Abbott Laboratories, Chicago, IL),.

*Details on the cell cycle arrested EREB2-5 cell line*

EREB2-5 cells are a non-classical LCL cell line with an estradiol-inducible EBV-latency III proliferation program due to an estrogen receptor fused to the EBNA2 viral transcription factor. Transfection, and hygromycin selection of EREB2-5 cells were performed as previously described (4). Estradiol starved EREB2-5 cells (cell cycle arrested) were treated with doxycycline (1ng/µL) for 48h to induce expression of the cDNA of interest.

*Antibodies*

Antibodies (Santa Cruz Biotechnology, CA, USA) used for supershift experiments are anti-RelA (F-6), anti-c-Rel (N), anti-p50 (E-10), and anti-Oct-2 (PT-2).

For western blot, the antibodies (Santa Cruz Biotechnology) used were : anti-RelA (F-6), anti-RelB (D-4), anti-c-Rel (N), anti-IκBα (FL), anti-IκBɛ (ab-22; Sigma-Aldrich, Saint Louis, USA) (all at 1/200); anti-TRAF1 (G-20) at 1/1000, anti-A20 (A-12) at 1/1000, anti-SAM68 (H-4) at 1/1000, and anti-αTubulin (B-5-1-2, Sigma-Aldrich) at 1/10 000.

*Apoptosis analysis*

For apoptosis analysis by ﬂow cytometry, cells were first stained with an anti-NGFR-APC antibody (BioLegend, San Diego, CA, USA) and then, double stained with AnnexinV-FITC (BioLegend, San Diego, CA, USA) and propidium iodide (Sigma-Aldrich, Saint-Louis, MO, USA) in cold PBS-CaCl2-MgCl2 (Invitrogen, Cergy-Pontoise, France). NGFRt positive apoptotic cells were analyzed on a FACS Calibur flow cytometer.

*SiRNA experiment*

SUDHL-4 cells (5.10^6^) were transiently transfected with 250 pmol siRNA targeting REL mRNA (siREL) or an siRNA control from Santa Cruz Biotechnology using Amaxa® Nucleofector® Technology with solution V (Lonza; Basel, Switzerland). siREL is a pool of 3 target-specific 19-25 nt siRNAs designed to knock-down REL gene expression. Percentage of dead cells was quantified by flow cytometry 48h after transfection on the SSC/FSC biparametric histogram.

**References**

1. Dubanet L, Bentayeb H, Petit B, Olivrie A, Saada S, de la Cruz-Morcillo MA, Lalloué F, Gourin M-P, Bordessoule D, Faumont N, et al. Anti-apoptotic role and clinical relevance of neurotrophins in diffuse large B-cell lymphomas. *Br J Cancer* (2015) **113**:934–944. doi:10.1038/bjc.2015.274

2. Hans CP, Weisenburger DD, Greiner TC, Gascoyne RD, Delabie J, Ott G, Müller-Hermelink HK, Campo E, Braziel RM, Jaffe ES, et al. Confirmation of the molecular classification of diffuse large B-cell lymphoma by immunohistochemistry using a tissue microarray. *Blood* (2004) **103**:275–282. doi:10.1182/blood-2003-05-1545

3. Dubois S, Viailly P-J, Mareschal S, Bohers E, Bertrand P, Ruminy P, Maingonnat C, Jais J-P, Peyrouze P, Figeac M, et al. Next-Generation Sequencing in Diffuse Large B-Cell Lymphoma Highlights Molecular Divergence and Therapeutic Opportunities: a LYSA Study. *Clin Cancer Res Off J Am Assoc Cancer Res* (2016) **22**:2919–2928. doi:10.1158/1078-0432.CCR-15-2305

4. Chanut A, Duguet F, Marfak A, David A, Petit B, Parrens M, Durand-Panteix S, Boulin-Deveza M, Gachard N, Youlyouz-Marfak I, et al. RelA and RelB cross-talk and function in epstein-barr virus transformed B-cells. *Leukemia* (2013) doi:10.1038/leu.2013.274

5. Wright G, Tan B, Rosenwald A, Hurt EH, Wiestner A, Staudt LM. A gene expression-based method to diagnose clinically distinct subgroups of diffuse large B cell lymphoma. *Proc Natl Acad Sci U S A* (2003) **100**:9991–9996. doi:10.1073/pnas.1732008100

6. Monti S, Savage KJ, Kutok JL, Feuerhake F, Kurtin P, Mihm M, Wu B, Pasqualucci L, Neuberg D, Aguiar RCT, et al. Molecular profiling of diffuse large B-cell lymphoma identifies robust subtypes including one characterized by host inflammatory response. *Blood* (2005) **105**:1851–1861. doi:10.1182/blood-2004-07-2947

7. Mareschal S, Ruminy P, Bagacean C, Marchand V, Cornic M, Jais J-P, Figeac M, Picquenot J-M, Molina TJ, Fest T, et al. Accurate Classification of Germinal Center B-Cell-Like/Activated B-Cell-Like Diffuse Large B-Cell Lymphoma Using a Simple and Rapid Reverse Transcriptase-Multiplex Ligation-Dependent Probe Amplification Assay: A CALYM Study. *J Mol Diagn JMD* (2015) doi:10.1016/j.jmoldx.2015.01.007

8. Chauzeix J, Pastoret C, Donaty L, Gachard N, Fest T, Feuillard J, Rizzo D. A reduced panel of eight genes (ATM, SF3B1, NOTCH1, BIRC3, XPO1, MYD88, TNFAIP3, and TP53) as an estimator of the tumor mutational burden in chronic lymphocytic leukemia. *Int J Lab Hematol* (2020) doi:10.1111/ijlh.13435
