# Supplementary material for: c-Rel Is the Pivotal NF-κB Subunit in Germinal Center Diffuse Large B-Cell Lymphoma: A LYSA Study
Source: Front Oncol. 2021 Apr 20;11:638897. doi: 10.3389/fonc.2021.638897 (PMC8095348; doi:10.3389/fonc.2021.638897)
Supplement: Supplementary file 5 [file DataSheet_1.zip › Supplementary Tables 1-2.DOCX]

**Legends of Supplementary Figures**

**Figure S1. Analysis flow from the test series of 48 DLBCL patients.** Good quality of mRNA and protein extracts was obtained from 44 and 32 patients respectively. Analysis of both mRNA expression and EMSA were done on 28 samples. Additional samples were analyzed for mRNA expression (n=16) or EMSA (n=4) only.

**Figure S2. Experimental design of gene expression analysis.** First, step (1) indicates unsupervised expression analysis of 237 genes previously found differentially expressed between DLBCL patients characterized by high or low c-Rel DNA-binding activity (EMSA): 74/202 (37%) and 90/233 (39%) patients with c-Rel GEP (*Gene Expression Profile*) from the GHEDI and LENZ series respectively. Secondly, step (2) corresponds to *REL* gene expression analysis (from 4 probesets): 100/202 (50%) and 113/233 (48%) patients with *REL* mRNA overexpression from the GHEDI and LENZ series respectively. Finally, step (1) + (2) designates patient samples with c-Rel signature meaning with both c-Rel GEP and *REL* mRNA overexpression: 60/202 (30%) and 69/233 (30%) patients with c-Rel signature from the GHEDI and LENZ series respectively.

**Figure S3. Overall survival (OS) of 137 DLBCLs from the GHEDI cohort classified according to c-Rel signature and EZB (with EZH2 mutations and BCL2 translocation) or other GCB genetic subtypes.** OS of EZB-DLBCL patients and GCB-DLBCL patients with c-Rel and non-c-Rel signature. The log-rank p-value is given within each graph.

**Figure S4.** **Mortality of GCB-DLBCL cell line under siRNA-REL transfection.** SUDHL-4 cells were transiently transfected with siRNA targeting REL mRNA (siREL) or siRNA control (siCtl). At 48h post-transfection, expression of c-Rel and αTubuline expressions **(A)** and percentage of dead cells **(B)** were analyzed by western blot and flow cytometry respectively. In panel B, Mortality fold change is the ratio to the mean of control conditions.

**Supplementary Tables**

**Table S1.** Clinical data from the test series.

| **Clinical Parameter** | **Total** |
| --- | --- |
| Gender M/F, n | 29/19 |
| Age (Median, range) | 65 (15 – 93) |
| Subtype, n (%) |  |
| Non-GCB | 18/48 (38%) |
| GCB | 30/48 (62%) |
| Nodal / Extranodal, n (%)l |  |
| Nodal | 31 (65%) |
| Extranodal | 17 (35%) |
| Adverse Pronostic Factors |  |
| Age > 60 years, n, (%) | 30/48 (62%) |
| Ann Arbor stage III-IV | 28/47 (60%) |
| LDH>Normal | 19/46 (41%) |
| Performans Status ≥ 2 | 13/44 (30%) |
| IPI |  |
| 0 - 2 | 26/45 (58%) |
| 3 – 5 | 19/45 (42%) |
| Treatment |  |
| R-chemotherapy | 39/48 (81%) |
| Chemotherapy without R  Other | 5/48 (11%)  4/48 (8%) |

**Table S2.** Clinical data from the GHEDI (Dubois) series.

| **Clinical Parameter** | **Total** |
| --- | --- |
| Gender M/F, n | 104/98 |
| Age (Median, range) | 61 (19 – 87) |
| Subtype, n (%) |  |
| ABC | 83 (41%) |
| GC | 85 (42%) |
| Other | 34 (17%) |
| Nodal / Extranodal, n (%)l |  |
| Nodal | 158 (78%) |
| Extranodal | 43 (21.5%) |
| Unknown | 1 (0.5%) |
| Adverse Pronostic Factors |  |
| Age > 60 years, n, (%) | 106 (52%) |
| Ann Arbor stage III-IV | 144 (71%) |
| LDH>Normal | 124 (61%) |
| Performans Status ≥ 2 | 34 (17%) |
| IPI |  |
| 0 - 2 | 103 (51%) |
| 3 - 5 | 99 (49%) |
| Treatment |  |
| R-chemotherapy | 188 (93%) |
| Chemotherapy without R | 14 (7%) |

**Table S3**. The HGU133 +2.0 Affymetrix Gene Chip microarray data set (https://www.ncbi.nlm.nih.gov/geo; accession number GSE87371) (1–4). LIMMA analysis was performed on the 12 959 most variable genes.

**Table S4.** c-Rel DNA-binding activity related genes, *i.e.* c-Rel gene expression profile (GEP). Analyzed by Affimetrix GEP of DLBCLs from the GHEDI cohort. A set of 343 probesets/237 genes with a fold change of two between high and low or negative c-Rel binding cases was selected after LIMMA analysis.

**Table S5.** Overlaps between the two branches of down and up-regulated genes of the c-Rel gene expression profile of the two clusterings from the GHEDI and Lenz series.

**Table S6.** Set of 424 differentially expressed genes (622 probesets) with a fold change of two between ABC and GCB-DLBCLs from the training series: 66 cases from the GHEDI cohort. This ABC-GCB GEP consisted of 177 (279 probesets) and 247 (343 probesets) up-regulated genes in ABC (ABC-up) and GCB (GCB-up) DLBCLs respectively. Genes of the ABC/GCB Wright’s predictor (5) are indicated.

**Table S7.** Overlap between c-Rel and ABC-GCB gene expression profiles (*i.e.* c-Rel AND ABC-GCB gene set) corresponding to 55 genes and 88 probesets.

**Table S8.** Classification of 137 DLBCLs from the GHEDI cohort according to genetic subtypes defined by Schmitz *et al* (6): BN2 (based on *BCL6* fusions and *NOTCH2* mutations), EZB (based on EZH2 mutations and BCL2 translocations), MCD (based on the co-occurrence of MYD88L265P and CD79B mutations), N1 (based on NOTCH1 mutations), “other ABC”, “other GCB”, and “other unclassified”.

**Bibliography**

1. Dubois S, Viailly P-J, Mareschal S, Bohers E, Bertrand P, Ruminy P, Maingonnat C, Jais J-P, Peyrouze P, Figeac M, et al. Next-Generation Sequencing in Diffuse Large B-Cell Lymphoma Highlights Molecular Divergence and Therapeutic Opportunities: a LYSA Study. *Clin Cancer Res Off J Am Assoc Cancer Res* (2016) 22:2919–2928. doi:10.1158/1078-0432.CCR-15-2305

2. Bobée V, Ruminy P, Marchand V, Viailly P-J, Abdel Sater A, Veresezan L, Drieux F, Bérard C, Bohers E, Mareschal S, et al. Determination of Molecular Subtypes of Diffuse Large B-Cell Lymphoma Using a Reverse Transcriptase Multiplex Ligation-Dependent Probe Amplification Classifier: A CALYM Study. *J Mol Diagn JMD* (2017) 19:892–904. doi:10.1016/j.jmoldx.2017.07.007

3. Jais J-P, Molina TJ, Ruminy P, Gentien D, Reyes C, Scott DW, Rimsza LM, Wright G, Gascoyne RD, Staudt LM, et al. Reliable subtype classification of diffuse large B-cell lymphoma samples from GELA LNH2003 trials using the Lymph2Cx gene expression assay. *Haematologica* (2017) 102:e404–e406. doi:10.3324/haematol.2017.166827

4. Dubois S, Tesson B, Mareschal S, Viailly P-J, Bohers E, Ruminy P, Etancelin P, Peyrouze P, Copie-Bergman C, Fabiani B, et al. Refining diffuse large B-cell lymphoma subgroups using integrated analysis of molecular profiles. *EBioMedicine* (2019) 48:58–69. doi:10.1016/j.ebiom.2019.09.034

5. Wright G, Tan B, Rosenwald A, Hurt EH, Wiestner A, Staudt LM. A gene expression-based method to diagnose clinically distinct subgroups of diffuse large B cell lymphoma. *Proc Natl Acad Sci U S A* (2003) 100:9991–9996. doi:10.1073/pnas.1732008100

6. Schmitz R, Wright GW, Huang DW, Johnson CA, Phelan JD, Wang JQ, Roulland S, Kasbekar M, Young RM, Shaffer AL, et al. Genetics and Pathogenesis of Diffuse Large B-Cell Lymphoma. *N Engl J Med* (2018) 378:1396–1407. doi:10.1056/NEJMoa1801445
